# Epstein-Barr virus induces adhesion molecule CD226 (DNAM-1) expression during primary B cell transformation into lymphoblastoid cell lines

**DOI:** 10.1186/1750-9378-7-S1-P31

**Published:** 2012-04-19

**Authors:** Lisa V Grossman, Pavel A Nikitin, Sandeep Dave, Jason P Tourigny, Micah A Luftig

**Affiliations:** 1 Department of Molecular Genetics and Microbiology, Center for Virology, Duke University Medical Center, Durham, NC, USA; 2 Duke Institute for Genome Sciences and Policy, Duke University, Durham, NC, USA

## 

Epstein-Barr virus (EBV), an oncogenic herpesvirus associated with Burkitt’s lymphoma and other AIDS-related B cell malignancies, transforms primary human B cells into lymphoblastoid cell lines (LCLs) *ex vivo*. As LCLs express viral gene products similar to those found in EBV-mediated cancers, LCLs provide a practical model for tumorigenesis. Previous unpublished findings from our lab indicate that LCLs constitutively express the adhesion molecule CD226 (DNAM-1), found on virtually all peripheral blood NK cells, T cells, and monocytes, but only a small subset (~3%) of B cells. Although CD226 is known to mediate T-cell differentiation and cytotoxicity, NK cell cytotoxicity, NKT cell apoptosis, and monocyte extravasion, CD226 function in B cells remains relatively unstudied. Biochemically, CD226 functions to support the interaction between the intracellular adhesion molecules LFA-1 and ICAM-1. Here, we demonstrate that EBV specifically induces CD226 expression in primary human B cells and EBV-negative B lymphoblasts during viral-mediated proliferation and outgrowth. EBV infection of primary B cells increased CD226 surface expression 5-fold during early proliferation and approximately 30-fold upon transformation into LCLs. EBV-converted Burkitt’s lymphoma cells constitutively express CD226, while EBV-negative B cell lymphomas do not. Additionally, we demonstrate that LMP-1, an EBV latency III membrane oncoprotein, induces CD226 expression in EBV-negative Burkitt’s lymphoma cells. Finally, we demonstrate that the NFκB pathway regulates CD226 expression. Indeed, B cell lymphomas with high NFκB activity (activated B cell-like diffuse large B-cell lymphomas) express CD226 at higher levels than B cell lymphomas with low NFκB activity (germinal center B cell-like diffuse large B cell lymphomas). As CD226 supports the interaction between LFA-1 and ICAM-1, which is critical to maintain the constitutive aggregation of EBV-transformed B cells, we propose that EBV-mediated induction of CD226 drives cell-cell contact ensuring B cell survival. These data suggest that CD226, a newly identified EBV-induced cell adhesion molecule, may play a key role in the pathogenesis of AIDS-associated and other B cell lymphomas.

